# Unique Cancer Migratory Behaviors in Confined Spaces of Microgroove Topography with Acute Wall Angles

**DOI:** 10.1038/s41598-020-62988-8

**Published:** 2020-04-09

**Authors:** Tomohiro Yaginuma, Keiichiro Kushiro, Madoka Takai

**Affiliations:** Department of Bioengineering, Graduate School of Engineering, The University of Tokyo, 7-3-1 Hongo, Bunkyo-Ku, Tokyo 113-8656 Japan

**Keywords:** Cellular motility, Breast cancer, Cell migration

## Abstract

In recent years, many types of micro-engineered platform have been fabricated to investigate the influences of surrounding microenvironments on cell migration. Previous researches demonstrated that microgroove-based topographies can influence cell motilities of normal and cancerous cells differently. In this study, the microgroove wall angle was altered from obtuse to acute angles and the resulting differences in the responses of normal and cancer cells were investigated to explore the geometrical characteristics that can efficiently distinguish normal and cancer cells. Interestingly, different trends in cell motilities of normal and cancer cells were observed as the wall angles were varied between 60–120°, and in particular, invasive cancer cells exhibited a unique, oscillatory migratory behavior. Results from the immunostaining of cell mechanotransduction components suggested that this difference stemmed from directional extensions and adhesion behaviors of each cell type. In addition, the specific behaviors of invasive cancer cells were found to be dependent on the myosin II activity, and modulating the activity could revert cancerous behaviors to normal ones. These novel findings on the interactions of acute angle walls and cancer cell migration provide a new perspective on cancer metastasis and additional strategies via microstructure geometries for the manipulations of cell behaviors in microscale biodevices.

## Introduction

Cells in the body are constantly interacting with the surrounding microenvironments such as the extracellular matrix (ECM) and other cells. Depending on the conditions of such microenvironments, cells are known to alter their functions including adhesion^1–3^, migration^4–6^, and differentiation^7^. Specifically, cell migration is one of the most important cell functions that plays an important role in various physiological phenomena, such as immune response^8^, tissue formation^9–11^, and cancer metastasis^12–14^. The interactions between migrating cells and the surrounding environment are extremely complicated, so in order to simplify and isolate such interactions, many types of analytical platforms have been fabricated and the influences of surrounding microenvironments on cell migration have been investigated by utilizing these platforms. These studies have reported that cell migration is affected by both chemical and physical environmental factors, such as the surrounding chemical gradient, surface chemistry, stiffness and surface topography^4,5,15–22^.

Conventionally, the above studies have been conducted on two-dimensional (2D) substrates. However, in recent years it has been found that the microscale three-dimensional (3D) topography on the substrate surfaces could induce unique behaviors of cells that are different from the 2D culture conditions, and furthermore drastically alter the cell motility^1,14,23–29^. Moreover, it has been found that the degree of influence of 3D topographies is different depending on the capability of each cell to sense and interact with the substrate material. For example, the invasiveness of breast cancer cells was markedly enhanced in 3D culture methods compared to conventional 2D culture methods, while other tumorigenic cancer cells and normal cells did not show the invasion in the same matrix^30^. In another example, the microfibrillar patterns mimicking the extracellular matrix morphology induced chemotaxis of specific brain cancer cells, which was not observed on 2D substrates^31^. Across these studies, invasive cancer cells have been found to behave distinctively by being trapped in a 3D microtopography. Depending on the surface properties of the surrounding 3D microtopographies, such as cell adhesiveness, pore size and stiffness, they exhibited different migratory modes^14,27^. Lamellipodium migration, lobopodium migration and amoeboid migration are representative migratory modes observed in the past, and are based on different migration mechanisms. In other words, the confinement into certain 3D microtopographies was found to induce such modes of cell migration, in a different manner from the macroscopic 3D matrices or 2D substrates. In addition, as the previous researches have demonstrated, cells could dramatically change their migratory behaviors according to the surrounding microscale topography, and dependent on the property of each cell type. These researches on the regulation of cell migration utilizing 3D topographies are crucial in not only understanding both the fundamental machineries of cells and various phenomena in the body, but also to provide the foundation for new technologies for the manipulation or separation of cells, which would lead to further applications for disease diagnosis or therapy^18,31,32^.

As one potential uses of substrate topographies in regulating cell migration, we have previously reported that microgroove-based topographies at the size scale of cells enhanced the cell motility and emphasize the motility differences between normal and cancer cells^33,34^. In addition, for normal cells, the topographical effects on cell migration were found to be more remarkable along 90° walls than >90° ones. It was suggested that this simple geometrical tissue culture system combining 2D bottom planes and wall planes partially induced certain cell migration modes reminiscent of those in 3D microtopographies, and the wall angle was a key factor. Therefore, in this research, the microgroove wall angle was altered from the range of obtuse to acute angles, and the differences in the responses of several types of cells, including metastatic cancer cells, to the microgroove topographies with different wall angles were investigated. Especially, the acute angle walls were expected to in part physically confine cells like 3D-culture and were expected to induce a unique cell migration modes in between 3D microtopographies and 2D flat surfaces. Although such well-defined microstructures containing sharp, acute angle walls do not exist in physiological systems, these studies would allow us to deepen our understandings of the effects of microgroove topographies on cell migration and lead to more effective cell regulation. Furthermore, it may lead to new insights into the fundamental cellular mechanisms in a physically confined space and may open up different avenues in utilizing microscale platforms for cell behavior analysis.

## Results and Discussions

### Fabrication of PDMS microgroove structure with various wall angles

**Figure 1:**
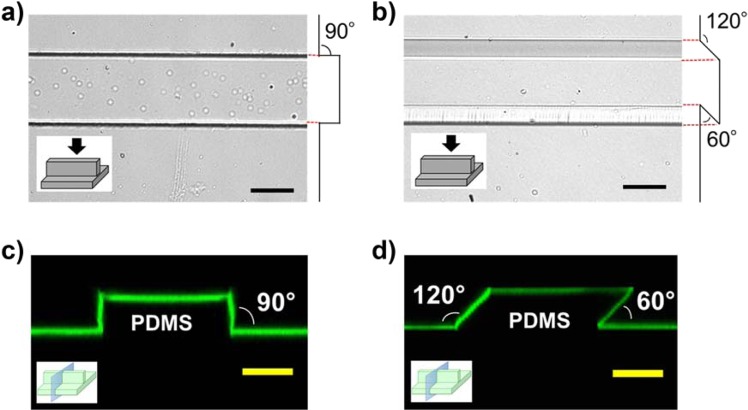
Bright-field image of the microgroove-based topographies with (**a**) a vertical wall and (**b**) a slanted wall. Confocal images of the cross sections of the microgroove-based topographies with (**c**) vertical wall and (**d**) slanted wall visualized by the adsorbed FITC-BSA. (Scale bar, 50 μm).

PDMS substrates with microgroove topographies containing different wall angles were fabricated by replica molding. To assess the fabricated topographies, bovine serum albumin (BSA) tagged with fluorescence was adsorbed on the surface of the substrates and the fabricated topographies were observed using a confocal microscope. From the 3D confocal imaging of the fluorescently labeled BSA, both the vertical and slanted microgroove structures were confirmed to be successfully fabricated (Fig. 1). As shown in the cross-sectional images of the microstructures, the slanted microgrooves had acute edges on one side and obtuse edges on the other side. By utilizing Ni-molds with such paired wall angles, microgroove structures with seven different wall angles (60, 70, 80, 90, 100, 110, and 120 degrees) were prepared for cell experiments.

### Cell motility on microgroove structures with various wall angles

**Figure 2:**
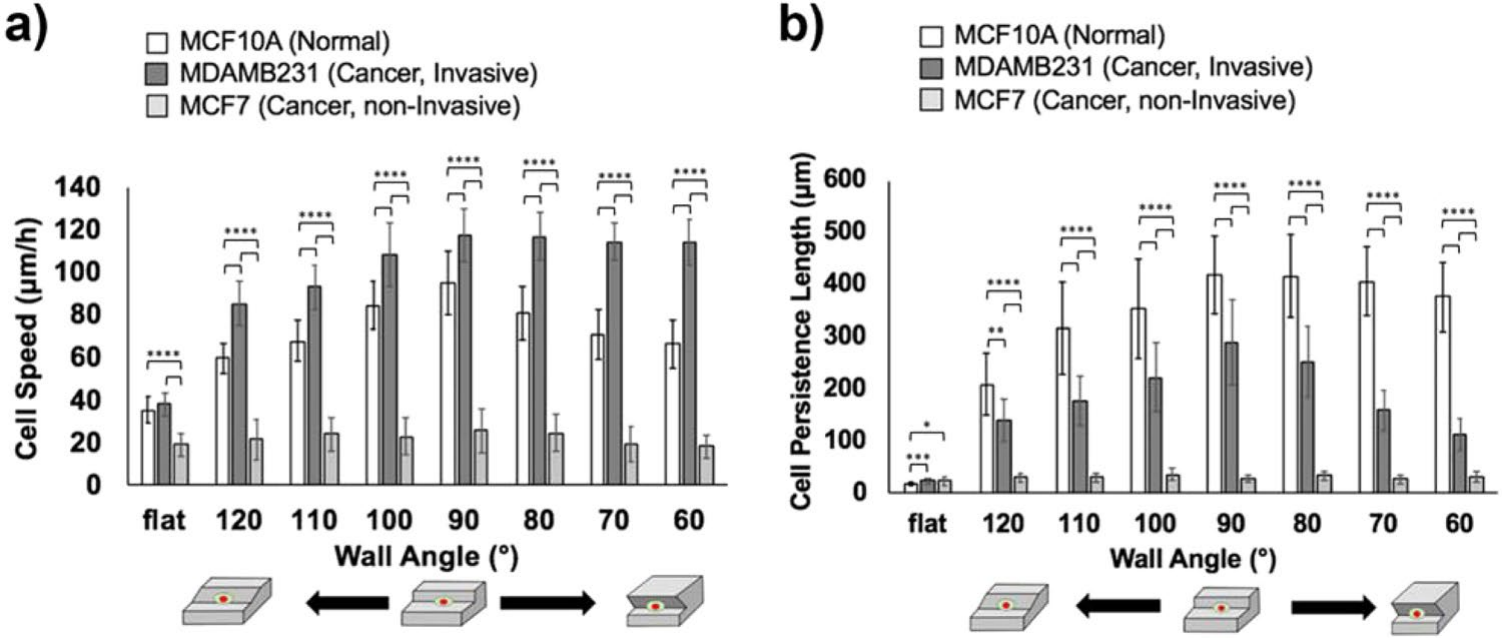
(**a**) Cell speed and (**b**) cell persistence length of MCF10A cells (normal), MDAMB231 cells (cancer, invasive) and MCF7 cells (cancer, non-invasive) along the edges of microgroove topographies with 60 to 120° walls and on the flat surface. (*0.01 < p < 0.05; **0.001 < p < 0.01; ***0.0001 < p < 0.001; ****p < 0.0001).

Three types of mammary epithelial cells, including breast cancer cell lines, were tested on the microgroove structures. Normal MCF10A cells, non-invasive MCF7 cancer cells and invasive MDAMB231cancer cells were seeded on the substrates with microgroove structures of different wall angles from 60 to 120°. Cell motilities on the microgroove topographies with different angles were evaluated from time-lapse images (Fig. 2). These were evaluated with 2 indices: speed and persistence. Speed was calculated as distance traveled by cells over time, while the persistence was an index for the directionality which was evaluated by measuring the distance that cells migrated until they changed their direction by >90 degrees. It should be noted that, especially for cells traveling along the microgroove structure, the absolute value of the distance traveled unidirectionally was measured as the persistence length, and consequently, speed was measured during this persistent movement, which had no reversal in direction.

From the results in Fig. 2, it was apparent that each cell type exhibited different responses to the microgroove topographies with varying wall angles. First, for normal MCF10A cells and invasive MDAMB231 cancer cells, there was no significant difference between the motilities of these cells on flat surfaces (normal cells: speed 36 ± 6 μm/h, persistence18 ± 2 μm; invasive cancer cells: speed 38 ± 6 μm/h, persistence 23 ± 4 μm). For 120–90° angles, normal and cancer cell motilities showed similar trends, where the velocities and persistence lengths of both types of cells increased as the wall angle decreased (normal cells: from 60 ± 7 to 95 ± 15 μm/h for speed, from 210 ± 60 to 419 ± 74 μm for persistence length; cancer cells: from 85 ± 10 to 118 ± 12 μm/h for speed, from 141 ± 41 to 290 ± 81 μm for persistence length) (Supplementary Information; Videos S1–S4). These results are consistent with our previous findings that cells along obtuse walls had difficulties in arranging the cytoskeleton along the microgroove edges, which led to the decrease of the motility parameter^33,34^. But when the wall angle was 60–90°, different trends were observed between normal and cancer cell motilities (Supplementary Information; Videos S5 and 6). In terms of cell speed, the speed of normal cells became smaller as the wall angle shifted from 90 to 60° (from 95 ± 15 to 66 ± 11 μm/h), while the speed of cancer cells did not change (from 118 ± 12 to 114 ± 11 μm/h), though it was overall larger than that of normal cells. In terms of persistence, the persistence length of normal cells did not change significantly as the wall angle shifted from 90 to 60° (from 419 ± 74 to 377 ± 65 μm), but the persistence length of cancer cells decreased (from 290 ± 81 to 113 ± 31 μm) and was smaller than that of normal cells overall. Regarding the persistence length of the cells, there was a characteristic difference in migration behaviors for each cell type. While normal cells continued unidirectional migration along the walls of microgroove topographies until they collided with other cells or entered the mitotic phase, cancer cells frequently reversed direction and thus oscillated along the wall, which was reflected on the decrease in persistence lengths (Supplementary Information; Videos S5 and S6).

For the non-invasive MCF7 cancer cells, motility did not change, regardless of the variations in the wall angle or the presence of the topography (speed: 18 ± 5 μm/h to 25 ± 10 μm/h, persistence length: 22 ± 9 μm to 36 ± 12 μm). It seemed to be caused by the lack of the 3D geometric recognition abilities of non-invasive cancer cells, which might be derived from the decreased interactions with underlying substrates compared to the other cell types (Supplementary Information; Video S7)^34^.

In addition to the motility parameters and related to the recognition of the 3D topography, there was a significant difference in the morphology of each cell type in response to various geometries. By evaluating the ratios of cells extending along the microgroove structure with unilamellar morphology (highly motile cells), it was found that there was a large difference between the normal and cancerous cell populations (Supplementary Information; Fig. S1). Along the vertical angle wall, about 70% of normal cells adopted the motile morphology but for 2 types of cancer cells the percentage of motile cells greatly decreased compared to the normal cells. This result indicated that 3D geometric recognition abilities of normal cells are much higher than cancer cells. Moreover, these results, in which invasive cancer cells with motile morphologies showed drastically higher motilities than non-invasive cancer cells, could reflect one of the heterogeneous features of tumors in which some cells display significantly higher motility over others, even though the average motility of the entire cell population is low due to the existence of the many non-motile cells^13,14,34^. At the same time, it was also demonstrated that the microgroove systems may be useful in differentiating the motile and non-motile cancer cells even in a heterogeneous cancer population.

Lastly, invasive cancer cells and normal cells showed similar trends in motility parameter when along microgroove topographies with obtuse and vertical angle walls (120-90°), but they showed completely different trends when along the microgroove topographies with acute angle walls (90-60°). In particular, invasive cancer cells showed the unique frequent reversal behaviors, and thus when along the acute angle walls, invasive cancer cells showed the migration behavior that can be clearly distinguished from other two cell types. These findings suggested that the acute wall angles could influence the cell migration more and induce the unique behaviors of different cell types.

### Immunostaining of cells and the analyses of extension behaviors and focal adhesions

To investigate the differences of cell motilities along acute angle walls, especially that between the normal MCF10A cells and invasive MDAMB231 cancer cells, key cell mechanotransduction components were visualized by immunostaining. The actin stress fibers were chosen to visualize the cytoskeletal reorganization in response to the microstructures, while vinculin was chosen to visualize the focal adhesions (FAs) to understand the cell migration direction and persistence. Consistent with the motility evaluations, we found that the extension behaviors and adhesion states were significantly different between normal and invasive cancer cells along the acute angle walls.

**Figure 3:**
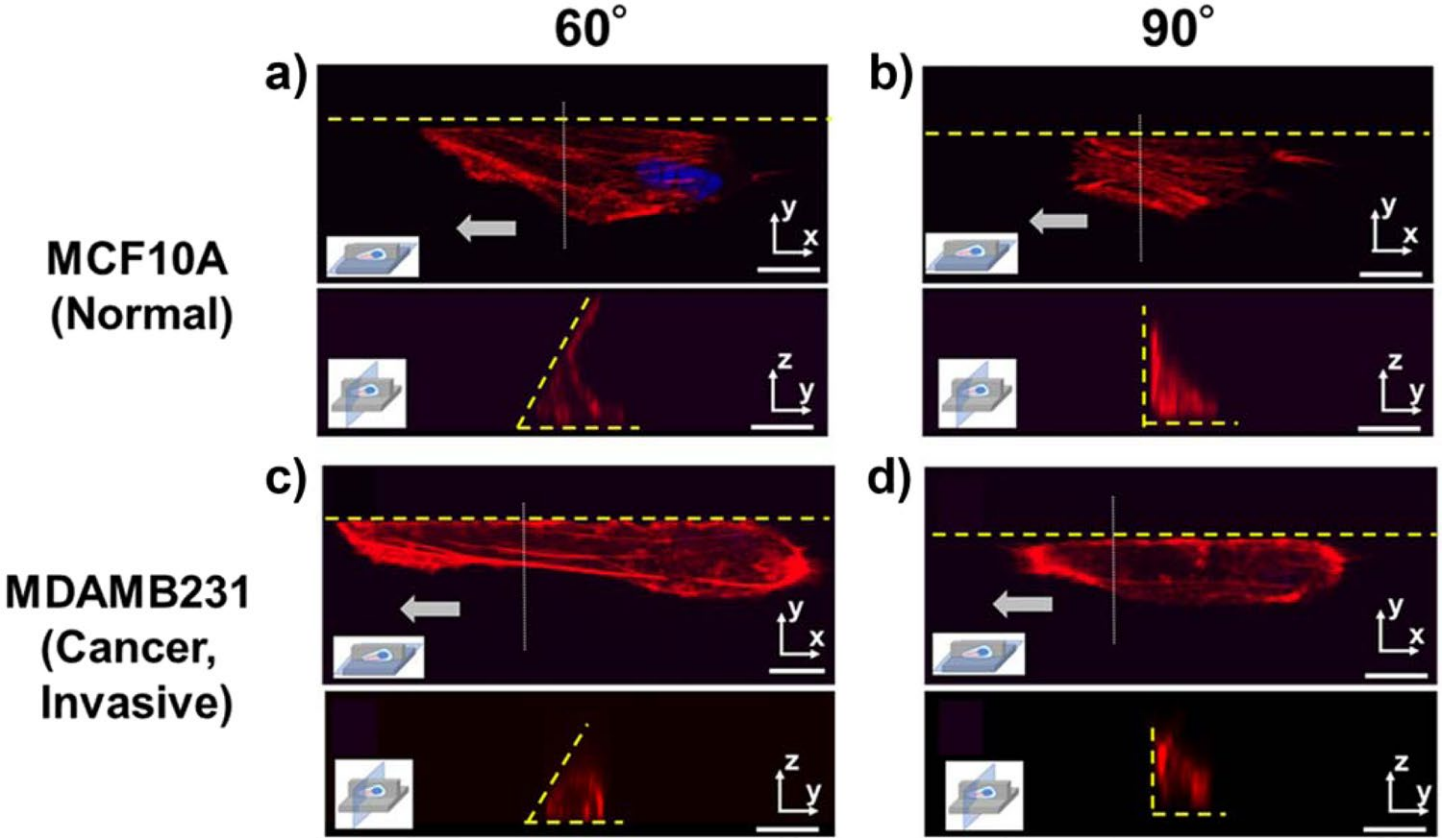
Immunostaining (63x) of actin (red) and nucleus (blue) of (**a**,**b**) MCF10A (normal) and (**c**,**d**) MDAMB231 (cancer, invasive) cells along the edge of microgroove topographies with 90° and 60° walls, with the same cell visualized on the X-Y planes and the Y-Z planes. Yellow dotted lines represent the microgroove wall boundaries. Gray arrows represent the direction of cell migration. (Scale bar: 10 μm).

First, the morphologies of the cells along the 90-60° walls were evaluated with confocal microscopy after staining the nucleus and actin filaments (Fig. 3). For both normal and invasive cancer cells, the extensions in the direction parallel to the wall were enhanced by the acute angle walls compared to the vertical walls. However, upon observing the cross sections of these cells, there was a striking difference in the manner of extension. For normal cells, as the wall angle decreased from 90° to 60°, those extensions in different directions of movement such as upward or lateral to the acute angle wall were enhanced due to the increased confinement (Fig. 3a,b). Meanwhile cancer cells further extended only in the direction of migration along the 90-60° walls (Fig. 3c,d). This difference in the extension behavior could explain the cause of the decrease in cell speed of normal cell along the <90° walls and corroborate the fact that the speed of invasive cancer cells was independent of the wall angle. As for the non-invasive MCF7 cancer cells, even along the wall of the microgroove topography, actin filaments were not aligned along the wall, unlike the other two cell types (Supplementary Information; Fig. S2). This result is consistent with the result that the motilities of non-invasive cancer cells did not change by the presence of the microtopography.

**Figure 4:**
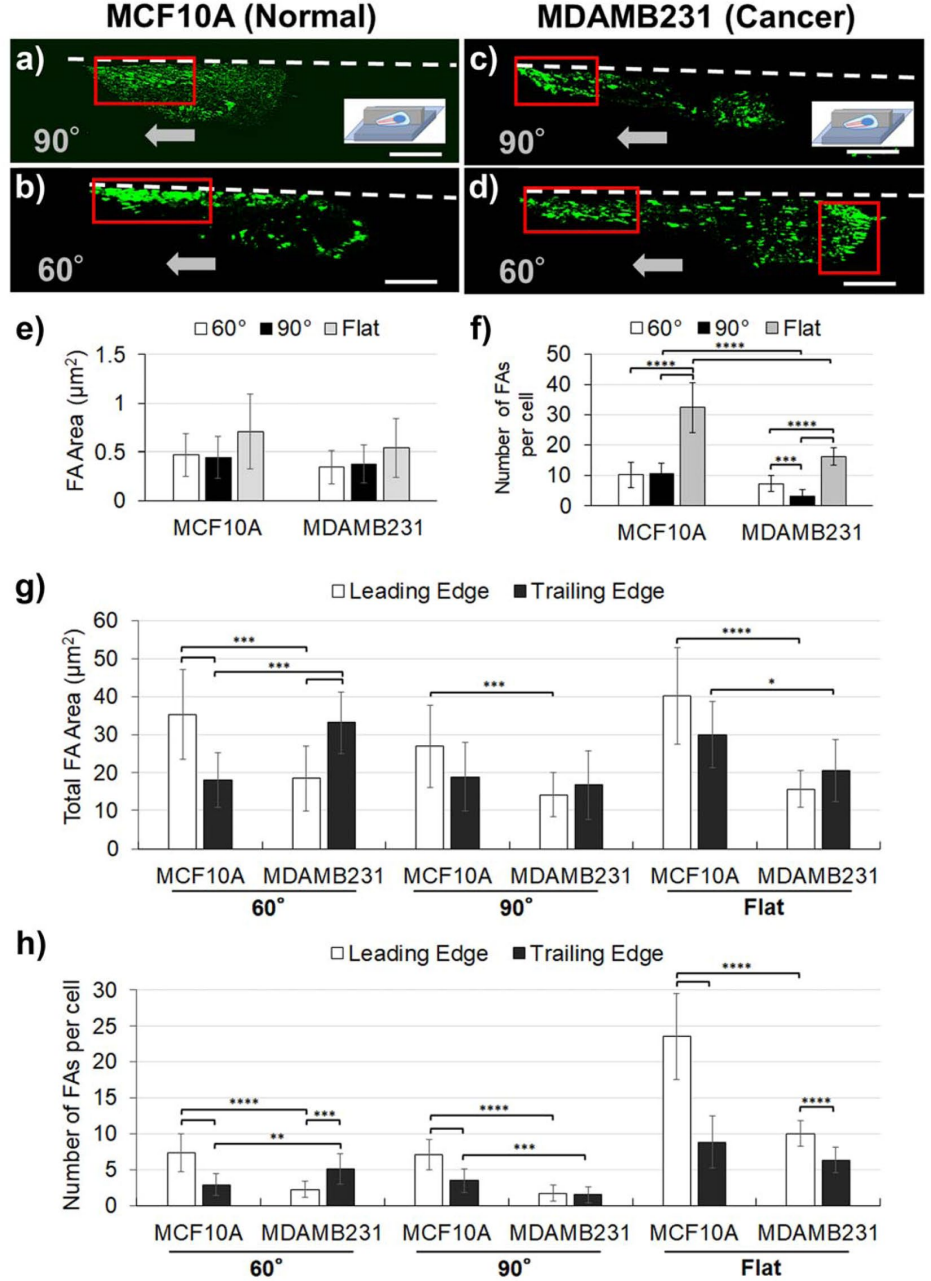
Immunostaining (63x) of vinculin (green) of (**a**,**b**) MCF10A (normal cells) and (**c**,**d**) MDAMB231 (cancer cells) along the edge of microgroove topographies with 90° and 60° walls. White dotted lines represent the microgroove wall boundaries. Gray arrows represent the direction of cell migration. Red boxes indicate dense areas of focal adhesions. Quantitative image analyses of focal adhesions for MCF10A (normal) and MDAMB231 (cancer, invasive) along the edge of microgroove topographies with 90° and 60° walls, and on the flat surface were performed. (**e**) Average focal adhesion areas and (**f**) the number of the matured focal adhesions (>0.5 μm^2^) contained in each single cell are shown. (**g**) Total focal adhesion areas and (**h**) the number of the matured focal adhesions (>0.5 μm^2^) contained in the leading edges and the trailing edges of normal and cancerous cells are shown. (Scale bar: 10 μm) (*0.01 < p < 0.05; **0.001 < p < 0.01; ***0.0001 < p < 0.001; ****p < 0.0001).

Then, to evaluate the adhesion states of cells, vinculin molecules were stained and also quantified through confocal microscopy images (Fig. 4). Vinculin is one of the main components of FAs, which represent the strength and directionality of cell adhesion^19,35^. For normal cells, FAs along the 60° and 90° walls and on the flat surface were found to be densely concentrated at the leading edge of the migrating cell (Fig. 4a,b). Those of invasive cancer cells along the flat surface showed a similar trend to the normal cells. However, regarding invasive cancer cells along the 60° and 90° walls, FAs appeared densely at the trailing edges as well as at the leading edges and that trend was more enhanced when the wall angle became smaller (Fig. 4c,d).

The FA properties, such as the number or the area of FAs, were also analyzed quantitatively from the obtained confocal images using ImageJ. In addition to these, the total FA area and the distribution of FAs between the leading edges and the trailing edges of the cells were analyzed, in which each cell was divided in half and FAs were separately counted for each side. The results indicated that, for the average area of FAs, there was no significant differences between the cells along the 60° and 90° walls, although FAs of normal cells were overall larger than ones of invasive cancer cells (Fig. 4e). Regarding the total number of FAs, the trends in the FA number (Fig. 4f) seemed to inversely correlate with the trends in persistence length (Fig. 2b), suggesting that FA number may be indicative of the persistence in cell migration. Further insights could be gained when FA analysis was performed separately on the leading edges and the trailing edges. First, when the total FA area of each edge were considered, it was found that in most cases, the leading edge had larger FA areas for the persistent normal cells, while such trend was not clearly observed for the invasive cancer cells, and even the opposite was true for cancerous cells in contact with the 60° walls (Fig. 4g). As for the distribution of the number of FAs of each edge, similar trends were observed with more significant differences. For normal cells, the number of FAs at the leading edges were more than twice as many as that at the trailing edges (Fig. 4h), consistent with their high persistence lengths. For invasive cancer cells, although the number of FAs at the leading edges were more than that at the trailing edges on the flat surface, FAs formed equally at leading and trailing edges along the 90° walls (Fig. 4h). Again, along the 60° walls the number of FAs at the trailing edges were significantly larger than at the leading edges, which may reflect the underlying FA reversal preceding the reversal of cell migration direction.

Taken together, most conditions where the cells displayed high persistent lengths were marked by both higher number and larger total FA areas at the leading edge compared to the trailing edge, while the frequent reversal behavior of the invasive cancer cells at the 60° walls were uniquely marked by the opposite trend of trailing edge dominance. Such underlying adhesion states of invasive cancer cells in the confined space of the acute angle wall may explain the frequent reversal of the migration directions, which cause the drastic decrease of the persistence length of invasive cancer cells along the acute angle walls. At the same time, these findings suggest that acute angle walls may be a useful geometrical feature to trap only the invasive cancers in place.

### Roles of myosin II in regulating cell motilities along acute angle walls

**Figure 5:**
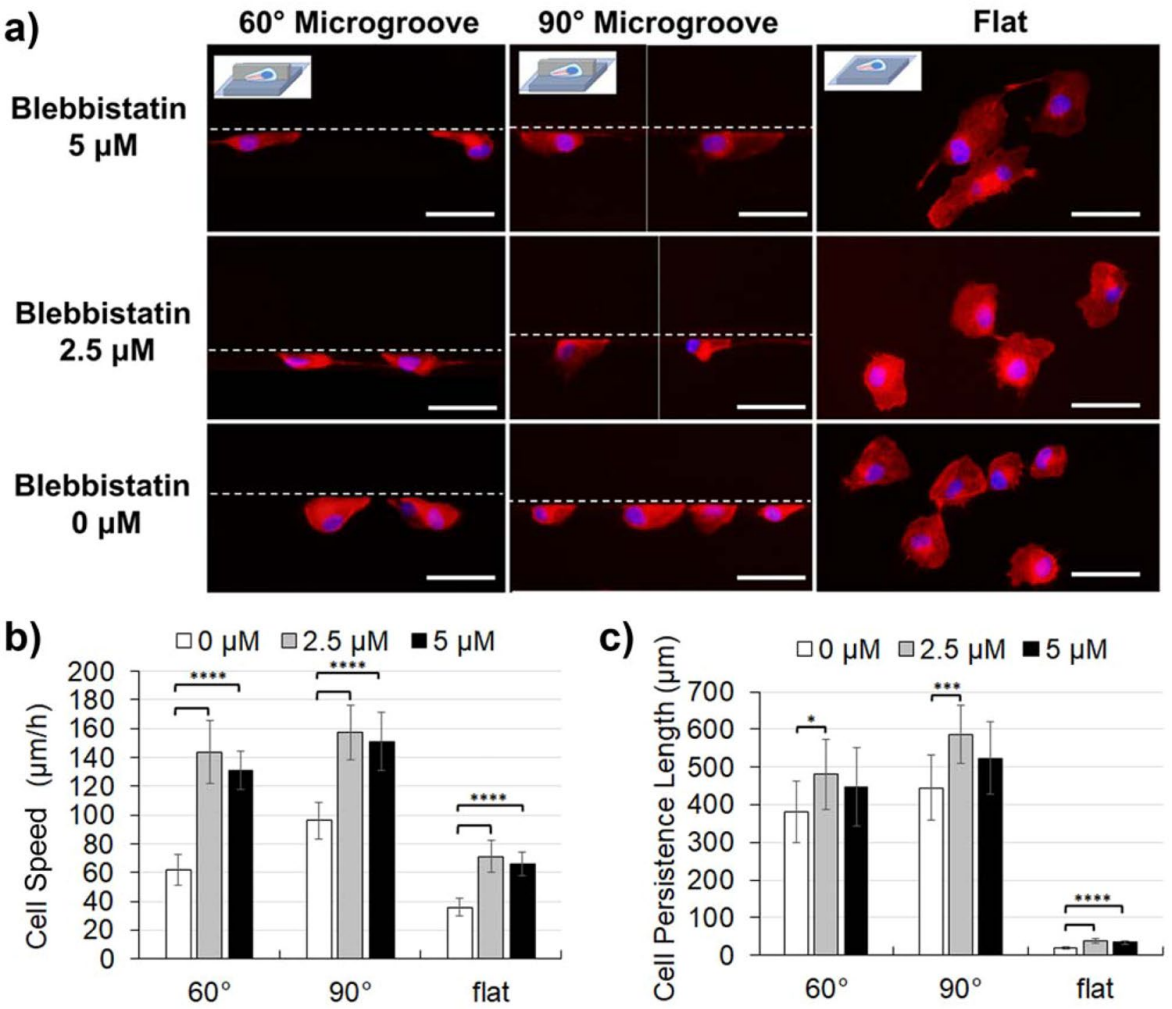
The morphologies and motilities of MCF10A cells (normal) treated with blebbistatin, a myosin II inhibitor. (**a**) Cell morphologies visualized by immunostaining of actin (red) and nucleus (blue) along the edge of microgroove topographies with 90° and 60° walls, or on the flat surface. Blebbistatin concentration was varied from 0 μM to 5 μM. White dotted lines represent the microgroove wall boundaries. (**b**) Cell speed and (**c**) cell persistence length of MCF10A cells treated with blebbistatin. (Scale bar: 50 μm) (*0.01 < p < 0.05; **0.001 < p < 0.01; ***0.0001 < p < 0.001; ****p < 0.0001).

**Figure 6:**
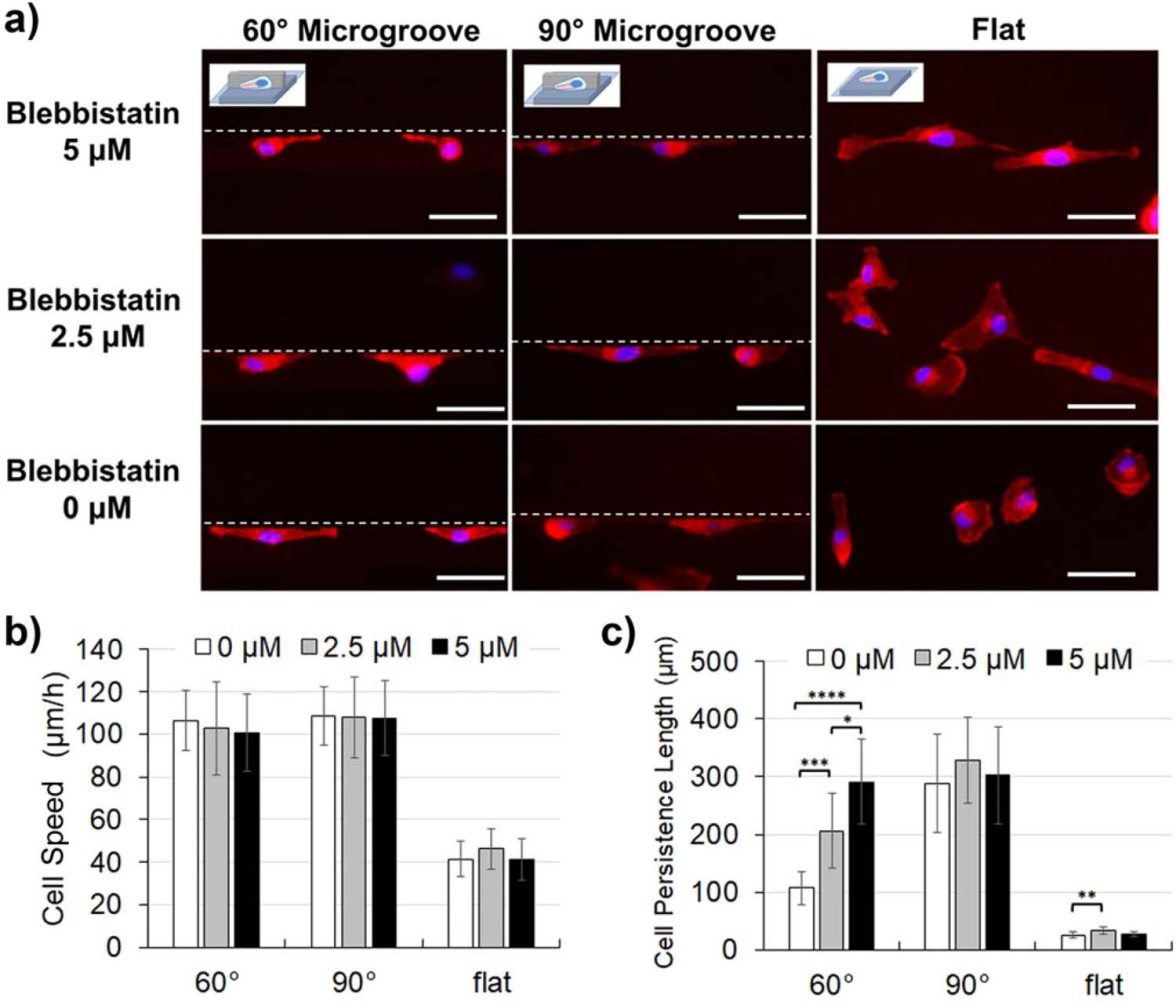
The morphologies and motilities of MDAMB231 cells (cancer, invasive) treated with blebbistatin, a myosin II inhibitor. (**a**) Cell morphologies visualized by immunostaining of actin (red) and nucleus (blue) along the edge of microgroove topographies with 90° and 60° walls, or on the flat surface. Blebbistatin concentration was varied from 0 μм to 5 μм. White dotted lines represent the microgroove wall boundaries. (**b**) Cell speed and (**c**) cell persistence length of MDAMB231 cells treated with blebbistatin. (Scale bar: 50 μm) (*0.01 < p < 0.05; **0.001 < p < 0.01; ***0.0001 < p < 0.001; ****p < 0.0001).

In order to prod the system further, the underlying mechanisms related to the difference in the motilities of normal and invasive cancer cells along acute angle walls were investigated. In particular, the focus was on the unique migratory mode of invasive cancer cells along acute angle walls from the perspective of migration machinery. The motilities of invasive cancer cells have been known to be greatly influenced by surrounding environments, and a physical confinement is one of the key factors influencing the cancer cell motility^14,27^. In a similar manner, microgroove topographies with acute wall angles may be perceived like a confined space by cells, possibly inducing the unique behavior of invasive cancer cells. Thus, factors relating to the cell migration in the confined space were investigated. In particular, myosin II has been known to be deeply involved in cancer cell migration within confined spaces and such cancer cell migration has been reported to be mainly driven by the contraction force generated by myosin II^14,27,36–38^. In other words, the activity of myosin, which has been correlated to the degree of the physical confinement felt by cells, may be considered as a tunable parameter to regulate the mode of motility. Using blebbistatin, a myosin II inhibitor, the relationship between the cell migration mode along the acute angle walls and myosin II activity was investigated. Morphologies and motilities of normal MCF10A cells (Fig. 5) and invasive MDAMB 231 cancer cells (Fig. 6) were evaluated after being seeded on the substrate with the microgroove topographies and treated with blebbistatin (concentration in growth medium: 0 μм, 2.5 μм, 5 μм).

First the cell morphologies were observed and it was found that blebbistatin treatment greatly changed cell morphology (Figs. 5a and 6a). With respect to normal cells, the cells spread broadly on a flat surface with blebbistatin treatment. When these cells were along the microgroove topography, the lamellipodial extensions became larger and the detachment of FAs at the rear end of the cells seemed less efficient. There was no remarkable difference between the normal cells along 60° and 90° walls. Next, for the invasive cancer cells, the cells spread broadly on a flat surface with blebbistatin treatment, similar to the normal cells. When invasive cancer cells were along the vertical walls, although there was no significant change in the morphology, FA detachment also seemed less efficient at the rear end of the cells. In contrary, however, when along the acute angle wall, the bidirectional stretching behavior was suppressed by the blebbistatin treatment and the cells extended unidirectionally along the wall, which was the morphology associated with high motility (Fig. 6a).

Quantitative evaluations of cell motilities under the influence of blebbistatin for normal and invasive cancer cells along 60° and 90° walls provided additional insights into the critical factors and mechanisms distinguishing these cells. First, for normal cells, blebbistatin treatment was found to greatly enhance both the cell speed and the persistence length. This trend was observed commonly in all conditions regardless of the presence or wall angles of microgroove topography. Interestingly, the motility parameters were more enhanced when the concentration of blebbistatin was 2.5 μм than 5 μм (Fig. 5b,c). To discuss the relationship between mobility of normal cells and blebbistatin treatment, the contribution of myosin II to normal cell migration should be considered. Generally, myosin II is well known to deeply contribute to cell contractility, but indirectly it is also involved in various mechanisms of cell migration, such as the formation of FA or the formation of actin stress fibers. Furthermore, suppression of myosin II has been reported to upregulate Rac1, which is a signaling factor that controls lamellipodial activity, and the activation of Rac1 is generally known to enhance cell motilities^39,40^. The motility enhancement by Rac1 activation is universal to the migration principle of normal cells and is consistent with the fact that the influence of blebbistatin treatment appeared similarly in all conditions regardless of the presence or the wall angle of microgroove topography. Furthermore, the morphology and the biphasic effects of blebbistatin on cell motility also suggest that at high concentrations, there may be not enough contraction to detach the FAs.

Next, the invasive cancer cells exhibited a different response to blebbistatin treatment compared to normal cells. It was found that the frequent reversal behaviors of invasive cancer cells along acute angle walls were neutralized by blebbistatin treatment (Supplementary Information; Video S8). This strongly suggested that the activity of myosin II may be deeply involved with the reversal behavior along acute walls. As a result of quantitative evaluation, it was found that the cell persistence length along the 60° wall increased as the blebbistatin concentration became higher (0 μм: 106 ± 28 μm; 2.5 μм: 206 ± 65 μm; 5 μм: 291 ± 73 μm), and interestingly, the influence of blebbistatin treatment did not appear in other geometrical conditions (Fig. 6b,c). This was a major difference between normal and invasive cancer cells, where for normal cells, the blebbistatin treatment enhanced the motility parameters at all conditions, while for invasive cancer cells, the influence of blebbistatin treatment appeared only in the persistence along the acute angle wall. This suggests that signaling and thus the mode of motility of invasive cancer cells along acute walls are indeed different from other conditions.

In addition, to prod the system in the opposite direction, the cells were treated with calyculin A, a myosin II enhancer. Normal MCF10A cells and invasive MDAMB 231 cancer cells were seeded on the substrate with the microgroove topographies and treated with calyculin A (concentration in growth medium: 0 nм, 0.1 nм). It was found that indeed the frequent reversal behaviors of invasive cancer cells worsened along acute angle walls and caused the persistence length to decrease dramatically, presumably due to the calyculin A treatment causing strong contractility (Supplementary Information; Fig. S3; Video S9). However, the reductions in persistence lengths were universal across both cell lines and across all topographies. It should also be noted that, consistent with previous studies^41^, the speeds of both cell types on flat surfaces were enhanced with calyculin A treatment, but such enhancements were not reflected on microstructured substrates, perhaps due to the limited lamellipodial extensions caused by calyculin A.

Lastly, in order to better understand the observed effects of blebbistatin and calyculin A, and more importantly the involvement of myosin contraction in the specific behaviors of invasive cancer cells in the confined space of the acute angle walls, the immunostaining of myosin IIA was performed for the normal MCF10A cells (Supplementary Information; Fig. S4) and the invasive MDAMB231 cancer cells (Supplementary Information; Fig. S5). Overall, it was confirmed that indeed blebbistatin reduced the activities of myosin IIA and thereby downregulated actin fiber formation, while calyculin A enhanced the activities of myosin IIA and thereby upregulated actin fiber formation. Importantly, it was demonstrated that myosin IIA distributions were highly consistent with the behaviors and characteristic morphologies of the cells in different conditions. For example, for the untreated MCF10A cells, myosin IIA was concentrated at the corners of the microstructure and parallel to the wall (Fig. S4), which is consistent with stable actin bundles forming parallel to the microstructures in previous studies^33^, and such focused contraction along the walls would explain the motility enhancements from topographical effects. As for the cancerous MDAMB231 cells along 60° walls that exhibited frequent reversals, myosin IIA was often strongly observed on both the leading and the trailing edges, which display the tug-of-war situation that ultimately lead to the reversal of migration direction (Fig. S5). This tug-of-war distribution was partially alleviated with the blebbistatin treatment, which caused the overall reduction in levels of myosin IIA and actin bundle formation, which would result in decreased traction forces that may trigger cells to reverse their migration direction. Thus, the key roles of myosin IIA in regulating cell motility behaviors along the small confinements of the acute angle walls has been further established. In fact, several previous studies have also reported that the aggregation and activation of myosin II were enhanced at the trailing edges of invasive cancer cells when they are trapped in spaces with strong physical confinement, such as non-degradable gels^14,36–38^. Taken together, the findings point to a similar mechanism of cell migration for acute angle walls and other confining systems, and it was demonstrated that the physical confinement due to the acute angle wall promoted the activation of myosin II at the trailing edges of invasive cancer cells, which caused the frequent reversal behaviors. Moreover, the findings unraveled the modulation of myosin II as a potential strategy to undo cancer-specific migration behaviors in physical confinements.

## Conclusion

In this paper, the relationship between the microgroove topography effects on cell migration and the wall angle was investigated. In summary, the differences among normal cells, non-invasive cancer cells, and invasive cancer cells became even greater when the wall angle of microgroove was in the acute range than in the obtuse range. It was observed that each cell type showed specific responses to the acute angle wall. Specifically, invasive cancer cells displayed a unique mode of motility where they frequently reversed the migration direction along the acute angle walls, while normal cells along the acute angle wall showed the similar migration behavior to the vertical wall but with only the speed decreased. The non-invasive cancer cells did not get affected by the topographies, similar to our previous studies. Immunostaining of actin fibers revealed the reason why cancer cells maintained the fast speed along the acute angle wall, while normal cell speed decreased. Moreover, immunostaining of vinculins revealed that the frequent reversal behaviors stemmed from the unique adhesion formations. These results also suggested that the lamellipodial extensions and the adhesion is a key driving force in topography-directed migration. Lastly, it was found that such unique behaviors of invasive cancer cells along the acute angle walls were extensively involved with the myosin II activities, with many similarities to cancer cells exposed to physical confinements. The findings from this study highlight the implications of the 3D confinement to cancer cell migration and deepen our understandings of the effects of microgroove topographies on cell migration, providing new geometrical tools and strategies to be applied to analysis and manipulation of cell behaviors in microscale platforms.

## Experimental Section

### Fabrication of PDMS microgroove structure with various wall angles

Microgroove structures (100-μm-wide and 40-μm-high; wall angles = 60, 70, 80, 90, 100, 110, and 120 degrees) were fabricated by casting polydimethylsiloxane (PDMS; Silpot 184 W/C; Dow Corning) onto the custom-made Ni-based mold, which was created in collaboration with Optnics Precision, Co. Briefly, silicon wafers were spin-coated with a negative photoresist (40-μm-thick), exposed to ultraviolet light at different angles through the chrome photomask, developed using a developer, sputtered with a conductive layer (300-nm-thick), and finally cast with an electroforming metal containing Ni to electroform a Ni-based mold (300-μm-thick) (Supplementary Information; Fig. S6). The PDMS elastomer base and the curing agent were mixed at the 10:1 weight ratio. PDMS substrates with microgroove structures were rendered cell adhesive by adsorbing fibronectin (Sigma) for 1 hr at 37 °C (10 μg/mL), and then blocked with 0.1% bovine serum albumin (Sigma) for 2 hr at 37 °C. To evaluate the fabricated substrates, PDMS substrates with microgroove topographies were immersed in fluorescein-isothiocyanate-conjugated bovine albumin (Sigma) solution (10 μg/mL) for 2 hr at 37 °C. Then surface topographies were observed using a confocal microscope (LSM510; Carl Zeiss) at 20× magnification.

### Cell culture

The invasive breast cancer MDAMB231 cells, non-invasive tumorigenic breast cancer MCF7 cells, and normal mammary epithelial MCF10A cells were used. MCF10A cells were cultured in growth medium composed of Dulbecco’s modified Eagle’s medium/Ham’s F-12 containing HEPES and L-glutamine (DMEM/F12, Invitrogen) supplemented with 5% horse serum (Invitrogen), 1% penicillin/streptomycin (Invitrogen), 10 μg/mL insulin (Sigma), 0.5 μg/mL hydrocortisone (Sigma), 20 ng/mL EGF (Peprotech) and 0.1 μg/mL cholera toxin (Sigma) and maintained under humidified conditions at 37 °C and 5% CO_2_. MDAMB-231 and MCF7 cells were cultured in growth medium composed of Dulbecco’s modified Eagle’s medium (DMEM, Invitrogen), supplemented with 10% fetal bovine serum (Invitrogen) and 1% penicillin/streptomycin (Invitrogen) and maintained under humidified conditions at 37 °C and 5% CO_2_. Cells were passaged regularly by dissociating confluent monolayers with 0.25% trypsin-EDTA (Invitrogen). Cells were passaged at 1:4 in growth medium. Blebbistatin (Sigma) dissolved in dimethylsulfoxide (DMSO) was used at 5 μM in growth media for the myosin II inhibition experiments. Calyculin A (Wako) dissolved in dimethylsulfoxide (DMSO) was used at 0.1 nM in growth media for the myosin II enhancement experiments.

### Time-lapse microscopy and motility quantification

Cells were seeded at 5 × 10^3^ cells/mL in growth medium on the micropatterned substrates at 5 × 10^3^ cells/mL at 37 °C and 5% CO_2_. After seeding for 24 hr, the incubated cells were imaged at 10× magnification every 5 min for 24 hr using CCM-1.4II D/C Cell Culture Monitoring System (ASTEC). In this system, the cells were maintained at 37 °C and 5% CO_2_ in a heated chamber with temperature and CO_2_ controller during time-lapse imaging. CCM Software 2.4.5.2 (ASTEC) was used for image processing. To track cell motility on micropatterned surfaces, the position of the leading edge of migrating cells was tracked using CCM and ImageJ software. Cell motility was evaluated by 2 indices: speed and persistence. To calculate the speed, the distance cells migrated for 0.5 hr was measured. It should be noted that time window during which cells changed their direction by >90 degrees were excluded from speed calculations because such actions drastically altered/changed the leading edge fronts used for the positional reference, thus causing high inconsistencies in measurements. The persistence was evaluated by measuring the distance that cells migrated until they changed their direction by >90 degrees. According to this rule, in terms of cells along the microgroove structure, the absolute value of the distance traveled unidirectionally along the microgroove was measured and used as persistence length.

For the evaluation of MCF10A and MDAMB231 cell motilities along the microgroove structure, isolated cells extending along the microgroove structure with unilamellar morphology and migrating independently of other cells were selectively chosen. In terms of MCF7 cells, which did not form the unilamellar morphology, isolated cells along the microgroove structure were targeted, regardless of the morphology. For evaluation of the motilities on flat substrates, isolated cells extended at a position 100 μm or more away from the microgroove were targeted. The unpaired, two-tailed Student’s t-test was used for statistical analysis. Differences were considered significant at p < 0.05. All the statistical analysis was performed using corresponding functions in Microsoft Excel.

### Immunostaining of nucleus, actin, vinculin and myosin IIA

Cells were fixed on 35 mm glass-bottomed dish (Iwaki) using 4% formaldehyde solution (Sigma) for 20 min at room temperature, permealized using 0.2% Triton X-100 (Amersham Biosciences) for 10 min at 4 °C, blocked with 0.1% bovine serum albumin (Sigma) overnight at 4 °C, and then incubated with dyes and antibodies. 4′,6-Diamidino-2-phenylindole, dihydrochloride (DAPI; Invitrogen) was incubated for 10 min to visualize the cell nucleus, Alexa Fluor 594 phalloidin (Invitrogen) was incubated for 1 hr to visualize the actin filaments, and primary anti-vinculin antibody (Abcam) or anti-non-muscle myosin IIA antibody (Abcam) was incubated overnight then tagged with secondary Alexa Fluor 488 goat anti-mouse IgG antibody (Invitrogen) or Alexa Fluor 488 goat anti-rabbit IgG antibody (Invitrogen), respectively, for 2 hr to visualize the target molecules. The fixed cells were visualized using a confocal microscope (LSM510; Carl Zeiss) at 63× magnification. Quantitative analysis of FAs was done by using ImageJ software.

## Supplementary information

Supplementary Information.

Video S1.

Video S2.

Video S3.

Video S4.

Video S5.

Video S6.

Video S7.

Video S8.

Video S9.
