# Supplementary material for: Unique Cancer Migratory Behaviors in Confined Spaces of Microgroove Topography with Acute Wall Angles
Source: Sci Rep. 2020 Apr 9;10:6110. doi: 10.1038/s41598-020-62988-8 (PMC7145876; doi:10.1038/s41598-020-62988-8)
Supplement: Supplementary file 1 — Supplementary Information. [file 41598_2020_62988_MOESM1_ESM.pdf]

Supplementary Information

Unique Cancer Migratory Behaviors in Confined Spaces of Microgroove Topography with Acute Wall Angles

Tomohiro Yaginuma, Keiichiro Kushiro, and Madoka Takai**

Supplementary Figures

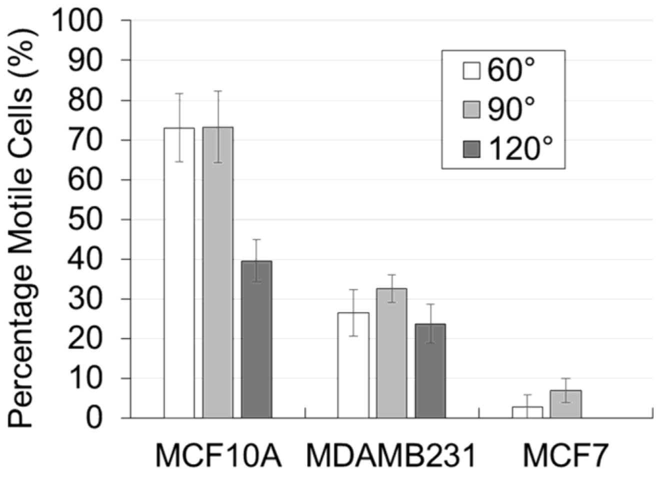

Figure S1. The percentage of cells with high-motile unilamellar morphology on the various wall angles for MCF10A cells (normal), MDAMB231 cells (cancer, invasive) and MCF7 cells (cancer, non-invasive) along the microgroove topographies.

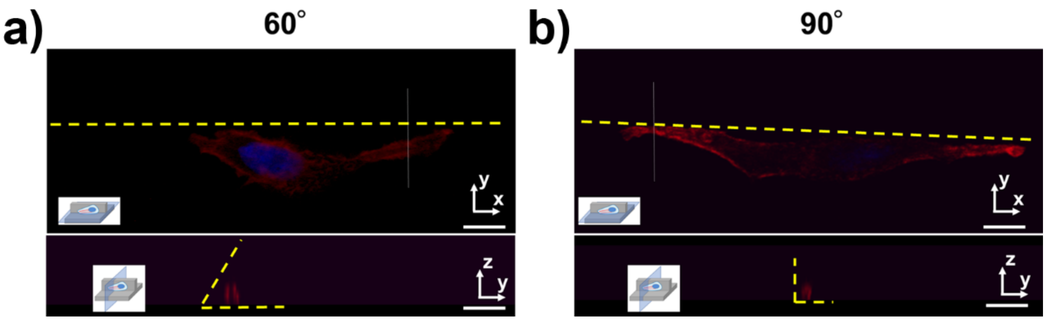

Figure S2. Immunostaining (63x) of actin (red) and nucleus (blue) of MCF7 (cancer, non-invasive) along the edges of microgroove topographies with (a) 60°and (b) 90° walls, with the same cell visualized on the X-Y plane and the Y-Z plane. Yellow dotted lines represent the microgroove wall boundaries. (Scale bar: 10 μm)

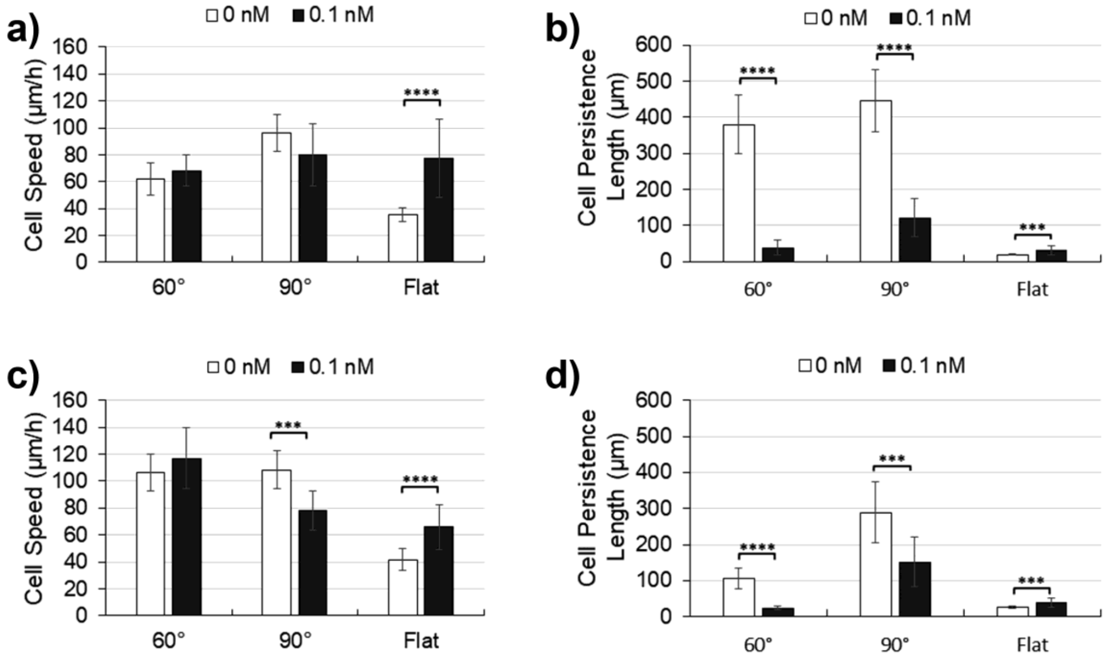

Figure S3. The motility analyses of (a,b) MCF10A (normal) and (c,d) MDAMB231 (cancer, invasive) treated with calyculin A, a myosin II enhancer. Calyculin A concentration was tested at 0.1 nM. (a,c) Cell speed and (b,d) cell persistence length of both cell types along the edge of microgroove topographies with 90° and 60° walls, or on the flat surface, are shown. (* : $0.01 < p < 0.05$; ** : $0.001 < p < 0.01$; *** : $0.0001 < p < 0.001$; **** : $p < 0.0001$)

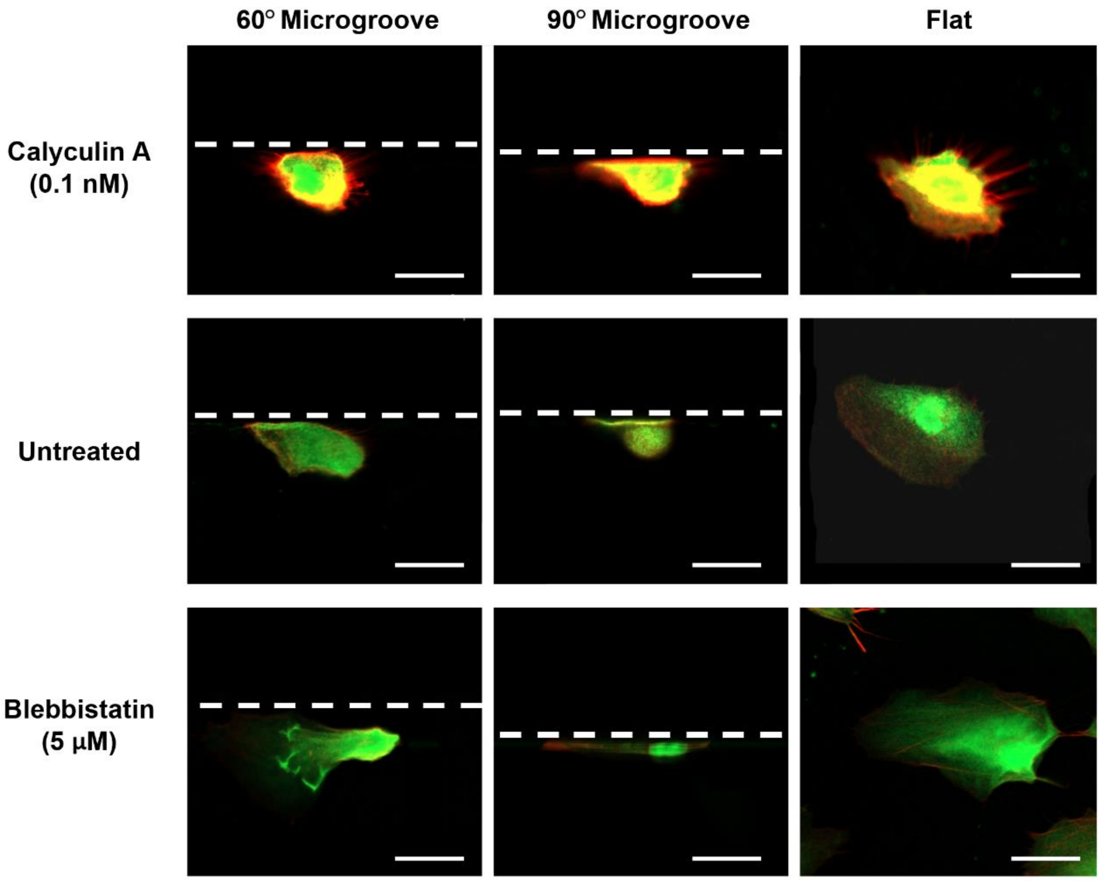

Figure S4. Myosin IIA distribution visualized via immunostaining of MCF10A cells (normal) treated with calyculin A, blebbistatin, or neither, along the edge of microgroove topographies with 90° and 60° walls, or on a flat surface. Cell were stained for myosin IIA (green) and actin (red). White dotted lines represent the microgroove wall boundaries. (Scale bar: 20 μm)

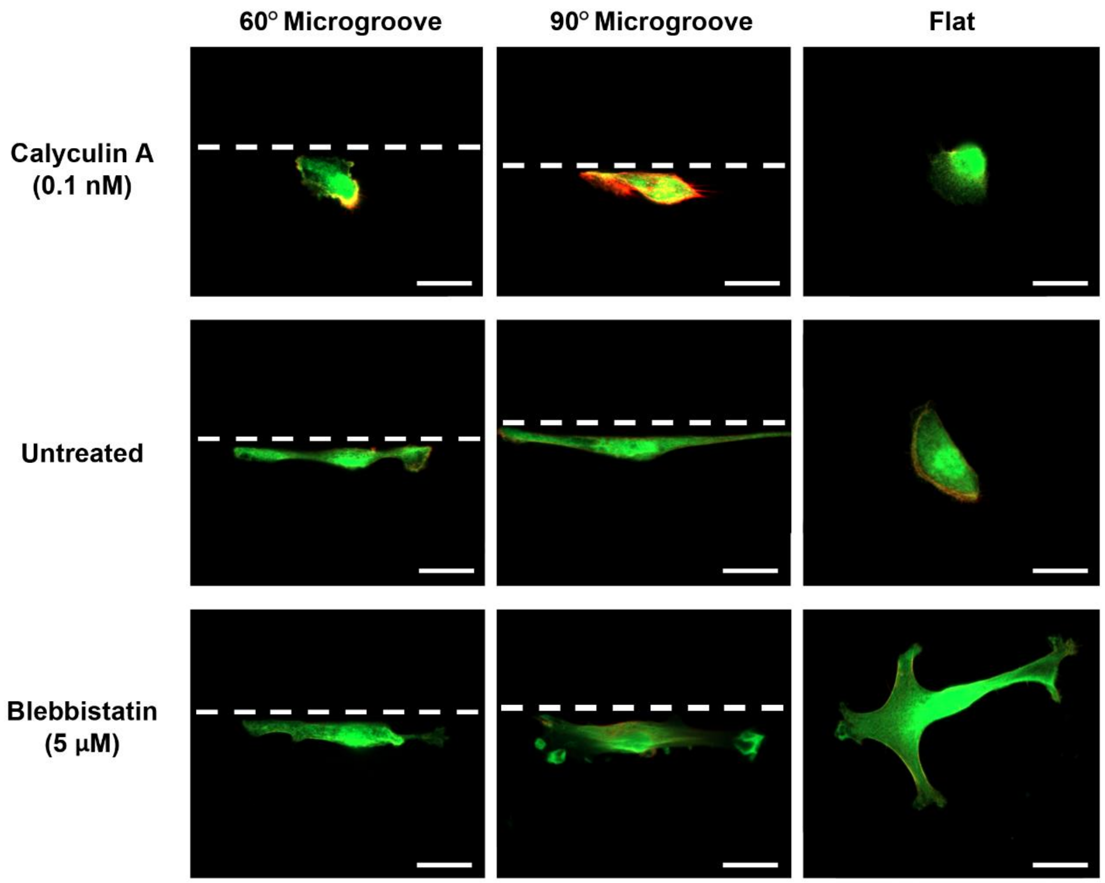

Figure S5. Myosin IIA distribution visualized via immunostaining of MDAMB231 cells (cancer, invasive) treated with calyculin A, blebbistatin, or neither, along the edge of microgroove topographies with 90° and 60° walls, or on a flat surface. Cell were stained for myosin IIA (green) and actin (red). White dotted lines represent the microgroove wall boundaries. (Scale bar: 20 μm)

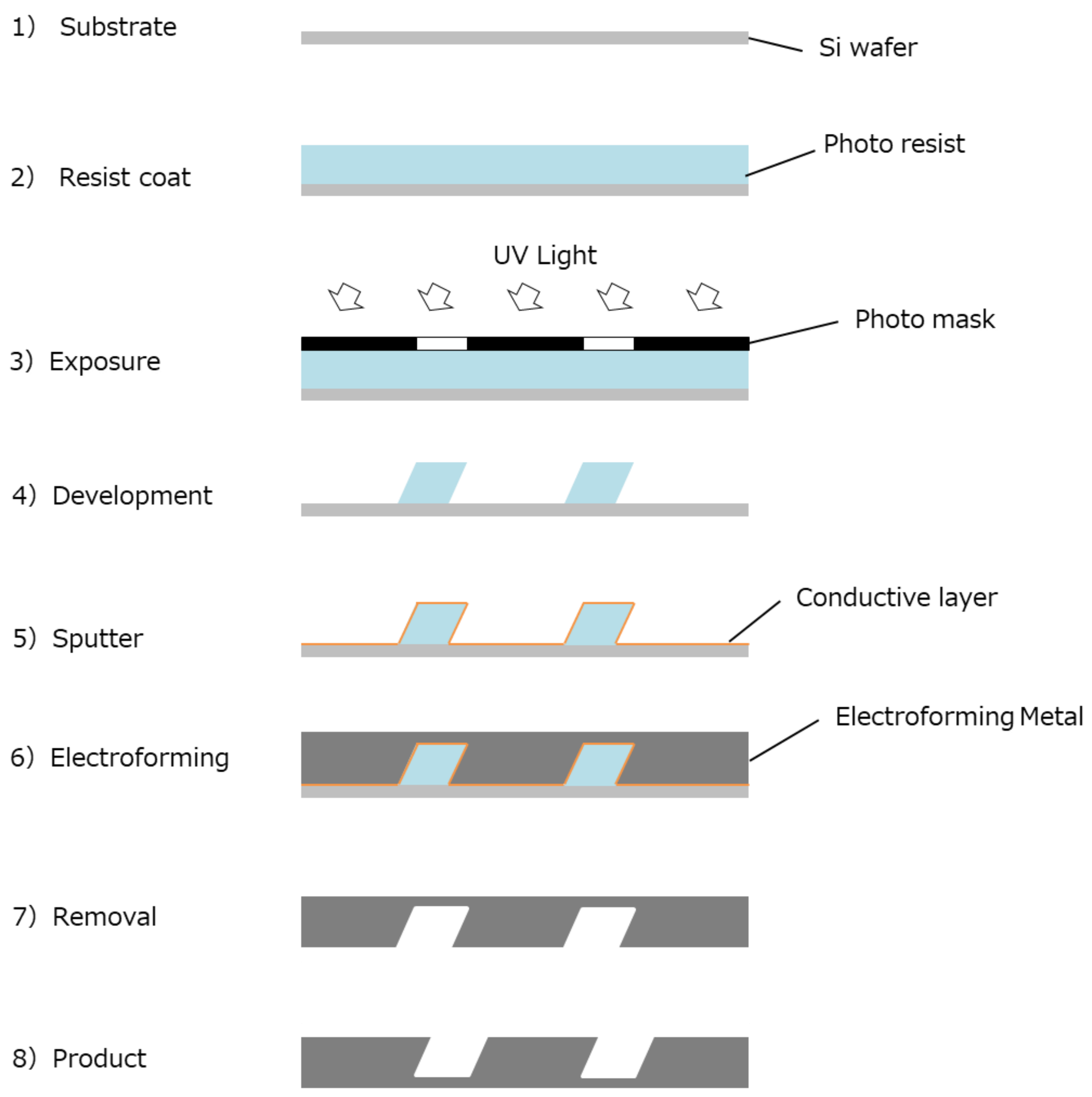

Figure S6. Schematic illustration of photoelectroformation of Ni-based molds with angled microgroove walls.

Supplementary Video Captions

Video S1. Normal cell (MCF10A) migration along the vertical angle wall (30 fps).

Video S2. Invasive cancer cell (MDAMB231) migration along the vertical angle wall (30 fps).

Video S3. Normal cell (MCF10A) migration along the obtuse angle wall (30 fps).

Video S4. Invasive cancer cell (MDAMB231) migration along the obtuse angle wall (30 fps).

Video S5. Normal cell (MCF10A) migration along the acute angle wall (30 fps).

Video S6. Invasive cancer cell (MDAMB231) migration along the acute angle wall (30 fps).

Video S7. Non-invasive cancer cell (MCF7) migration along the acute angle wall (30 fps).

Video S8. Cell migration of invasive cancer cells (MDAMB231) treated with 5 μM blebbistatin along the acute angle wall (30 fps).

Video S9. Cell migration of invasive cancer cells (MDAMB231) treated with 0.1 nM calyculin A along the acute angle wall (30 fps).
